# Suicide and suicidal behavior in the gulf cooperation council countries: a Systematic Review of behavioral patterns, sociocultural determinants, and structural vulnerabilities

**DOI:** 10.3389/fpsyt.2026.1857489

**Published:** 2026-06-26

**Authors:** Ahmed Mohammed Alasiri, Ahmed Nasser Alwulaii

**Affiliations:** Social Studies Department (Sociology), King Saud University, Riyadh, Saudi Arabia

**Keywords:** behavioral determinants, cultural stigma, GCC, gulf cooperation council, migrant workers, social integration, suicidal behavior, suicide

## Abstract

Suicide and suicidal behavior in the Gulf Cooperation Council (GCC) countries remain underrepresented in empirical research, largely due to cultural stigma, legal prohibitions, and systematic underreporting. The behavioral and sociocultural determinants of suicidal behavior in these contexts—where rapid modernization intersects with Islamic normative frameworks and large-scale labor migration—have received particularly limited systematic attention. This review synthesizes available evidence on the prevalence, behavioral patterns, risk factors, and structural determinants of suicidal behavior across Bahrain, Kuwait, Oman, Qatar, Saudi Arabia, and the United Arab Emirates. Following PRISMA 2020 guidelines, we searched PubMed, Scopus, ScienceDirect, Web of Science, and Google Scholar for primary research published between 2000 and 2025. Fifty studies (34 GCC-specific and 16 contextual/comparative) met the inclusion criteria. Methodological quality was appraised using the Newcastle–Ottawa Scale, JBI checklists, the Mixed Methods Appraisal Tool, and the AACODS checklist. The review protocol was registered on the Open Science Framework (DOI: 10.17605/OSF.IO/RZXY7; URL: https://osf.io/rzxy7/). The findings were organized into six dimensions: (1) suicide prevalence, risk factors, and associated behaviors; (2) cultural and religious influences; (3) mental health of specific at-risk populations; (4) healthcare and support services; (5) migrant worker vulnerabilities; and (6) research trends. Reported suicide rates in the GCC range from 1.5 to 4.2 per 100,000 but likely underestimate the true prevalence due to forensic misclassification and stigma. Migrant workers constitute a high-risk subgroup, with suicidal ideation reaching 68% among those facing acute financial distress. Islamic religiosity is associated with lower suicide mortality but simultaneously deters help-seeking by stigmatizing mental distress. Interpreted through a Durkheimian lens, these findings position suicidal behavior in the GCC as a social fact shaped by weakened social integration and normative fragmentation under rapid modernization. Effective prevention requires culturally adapted behavioral interventions that integrate labor welfare reforms with community-based mental health strategies.

## 1 Introduction

The World Health Organization estimates that over 720,000 people die by suicide annually, with approximately 20 nonfatal attempts for every death (1). Despite extensive global research, the Gulf Cooperation Council (GCC) countries—Bahrain, Kuwait, Oman, Qatar, Saudi Arabia, and the United Arab Emirates—have attracted comparatively little empirical attention, notwithstanding their distinctive sociocultural, religious, and economic conditions (2). Rapid modernization, large migrant worker populations, and Islamic normative frameworks shape both the behavioral expression of suicidal distress and the social structures that mediate risk and help-seeking in ways that diverge fundamentally from Western contexts (3). Therefore, understanding suicidal behavior in the GCC requires analytical frameworks that foreground these structural and sociocultural determinants rather than relying solely on clinical–individual models.

Prior regional syntheses have been limited in scope. Wakim et al. (4) conducted a bibliometric analysis of suicide research in the MENA region without synthesizing substantive findings. Alherz et al. (5) published a systematic review of suicide patterns in the GCC, considering 15 studies from two databases, focused primarily on demographic characteristics, methods, and risk factors. That review did not examine vulnerable subpopulations (e.g., migrant workers, healthcare professionals), did not analyze the dual role of religious norms as both protective and risk-enhancing, did not evaluate healthcare service utilization, and did not include a formal quality assessment of included studies.

Beyond GCC-focused reviews, recent regional scholarship has begun to address related dimensions of suicidal behavior in Arab and Muslim-majority contexts. Arafat et al. (6) compiled country-level evidence across Muslim-majority countries, including several GCC states, and highlighted the interplay between Islamic legal frameworks, social stigma, and gaps in epidemiological surveillance. Using a large online screener (N = 23,201), Daouk et al. (7) reported that 78.9% of Arabic-speaking respondents endorsed suicidality, with women and younger individuals appearing at disproportionate risk. Evidence from Iraq further illustrates some of the recurring regional concerns. Younis and Lafta (8), in a systematic review, estimated the suicide rate at 1.7 per 100,000 and identified underreporting, self-hanging, and youth vulnerability as dominant patterns. More recently, Younis et al. (9) found that 29.6% of psychiatric outpatients in Baghdad reported suicidal ideation, with mood disorders and substance use emerging as key correlates. Taken together, these studies show that regional evidence is expanding, but it remains uneven and only partially applicable to the GCC. GCC-specific evidence therefore remains thin compared with the broader MENA and Muslim-majority literature.

The present review addresses these gaps using a broader search strategy (five databases), a larger evidence base (50 studies), and a six-dimensional analytical framework integrating epidemiological, cultural, structural, service-related, population-specific, and migrant-focused dimensions. The GCC hosts millions of expatriate workers who, in some states, constitute a demographic majority (10). Many face conditions—contractual insecurity, social isolation, restricted healthcare access—that elevate vulnerability to suicidal behavior (11). Cultural stigma and legal penalties for suicide attempts further suppress reporting, creating gaps that standard surveillance fails to capture (12).

This review addresses four research questions: (1) What are the epidemiological prevalence rates and demographic patterns of suicidal behavior in the GCC? (2) How do Islamic religiosity and regional cultural norms function simultaneously as protective factors and barriers to help-seeking? (3) What structural determinants shape vulnerability among at-risk populations in the GCC, particularly migrant workers, healthcare professionals, and other marginalized groups? (4) What gaps in mental health services and crisis intervention infrastructure limit effective suicide prevention in the region?

## 2 Materials and methods

### 2.1 Protocol and registration

This review adheres to the PRISMA 2020 guidelines (13). The review protocol was prospectively registered on the Open Science Framework (DOI: 10.17605/OSF.IO/RZXY7). The completed PRISMA 2020 checklist is provided in **Supplementary Material S1**.

### 2.2 Search strategy

We searched five electronic databases: PubMed, Scopus, ScienceDirect, Web of Science, and Google Scholar. The search integrated controlled vocabulary (MeSH terms in PubMed) and free-text keywords. The initial search (January–March 2024) covered records from January 2000 to December 2023, followed by a supplementary update (January 2026) to incorporate 2024–2025 publications.

The core Boolean search string implemented in PubMed was as follows:

*(“suicide” OR “suicidal behavior” OR “suicidal ideation” OR “self-harm” OR “attempted suicide”) AND (“Gulf Cooperation Council” OR “GCC” OR “Bahrain” OR “Kuwait” OR “Oman” OR “Qatar” OR “Saudi Arabia” OR “United Arab Emirates” OR “UAE”)*

Database-specific syntax adaptations and exact search parameters are detailed in **Supplementary Material S2**. The search was supplemented by manually reviewing the reference lists of the included studies and high-impact regional reviews.

### 2.3 Analytical framework

The synthesis is organized around six dimensions. Four are substantive and correspond directly to the research questions: (1) suicide prevalence, risk factors, and associated behaviors; (2) cultural and religious influences; (3) mental health of specific at-risk populations, including migrant workers and healthcare professionals; and (4) healthcare and support services. Two additional dimensions provide contextual grounding: (5) migrant worker vulnerabilities, as a cross-cutting structural theme, and (6) research trends, encompassing temporal and thematic distribution, geographic coverage, and methodological approaches across the evidence base.

### 2.4 Eligibility criteria

Eligibility criteria followed the PICOS framework. The population comprised citizens, expatriates, and migrant workers residing in GCC countries, with no restrictions on age, gender, or clinical status. Studies of GCC nationals or GCC-relevant populations outside the region were considered only when they provided direct contextual or comparative relevance to the review. The exposure domain included reported risk factors, structural determinants, and behavioral correlates of suicidal behavior, including psychiatric morbidity, labor conditions, cultural stigma, and religious norms. No comparator was required, and studies with or without comparison groups were eligible. Outcomes included suicide mortality, suicide attempts, suicidal ideation, deliberate self-harm, and related behavioral or clinical outcomes. Eligible study designs were original empirical quantitative, qualitative, or mixed-methods studies published in English between 2000 and 2025. We excluded non-empirical records, duplicates, retracted articles, and studies on suicide terrorism or politically motivated self-destruction, as these fall outside the conceptual scope of clinical suicidology. Comparative or contextual studies conducted outside the GCC are marked with an asterisk (*) in the taxonomy tables.

### 2.5 Selection process and reliability

The systematic database search yielded 465 records (PubMed, *n* = 187; Scopus, *n* = 126; Web of Science, *n* = 89; ScienceDirect, *n* = 42; Google Scholar, *n* = 21). Following duplicate removal (*n* = 15) via Rayyan software (14), 450 records underwent title and abstract screening, of which 383 were excluded. Full-text evaluation of the remaining 67 articles led to the exclusion of 33 studies: 15 for focus misalignment or insufficient empirical data, 2 for addressing suicide terrorism, and 16 for not meeting inclusion criteria upon detailed review. This process yielded 34 GCC-specific primary studies from the database pathway.

**Figure 1:**
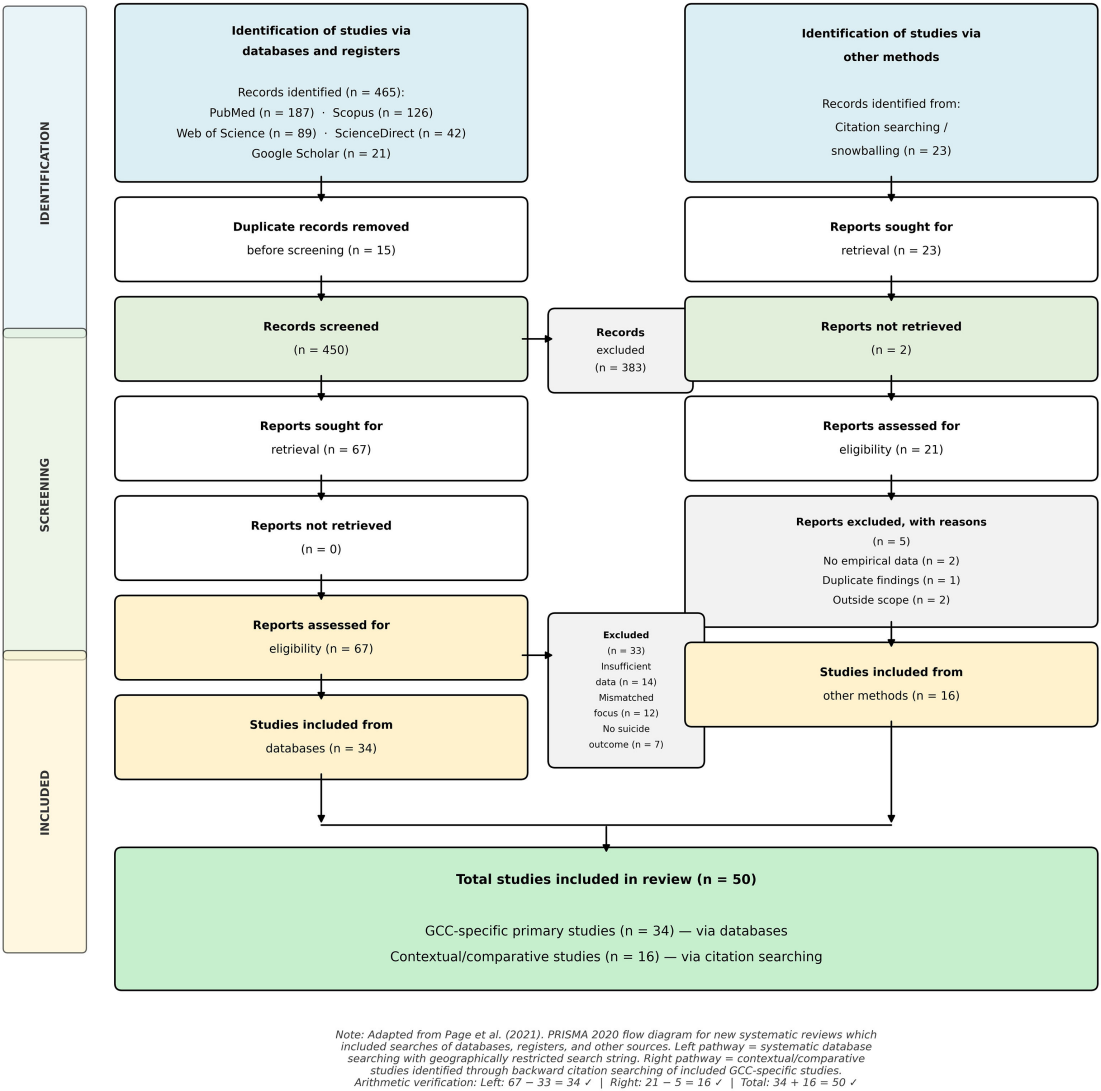
PRISMA 2020 flow diagram (*N* = 50). Left pathway: studies identified through systematic database searching. Right pathway: contextual/comparative studies identified through backward citation searching of included GCC-specific studies. Adapted from Page et al. (13).

An additional 16 contextual/comparative studies were identified through backward citation searching of the included GCC-specific studies and manual reference review. These were retained when they provided essential epidemiological benchmarking or addressed migrant-sending populations directly relevant to GCC contexts. The final synthesis comprises 50 studies (34 GCC-specific and 16 contextual/comparative). Two reviewers independently screened all records; inter-rater agreement reached 91% (κ = 0.84), with disagreements resolved through consultation with a third reviewer. **Figure 1** presents the PRISMA 2020 flow diagram.

### 2.6 Data extraction

Data were extracted using a standardized template capturing authorship, publication year, country, study design, sample characteristics, primary outcomes, and relevant sociocultural variables. A primary reviewer completed the extraction, with a second reviewer verifying all entries. This approach represents a pragmatic deviation from independent dual extraction, a limitation addressed in Section 4.

### 2.7 Risk of bias assessment

Methodological quality was appraised using design-specific validated instruments. Observational and cross-sectional studies (*n* = 29) were assessed via an adapted Newcastle–Ottawa Scale (NOS). Qualitative studies (*n* = 9) were evaluated using the JBI Critical Appraisal Checklist. Mixed methods studies (*n* = 8) were assessed with the Mixed Methods Appraisal Tool (MMAT v.2018). Non-empirical contextual papers (*n* = 4) were appraised using the AACODS Checklist. **Table 1** summarizes the results.

**Table 1:** Summary of risk of bias assessment results.

Tool used	Low risk	Some concerns	High risk	Total
NOS—Cross-sectional & Retrospective (15)	16	10	3	29
JBI Checklist—Qualitative (16)	5	3	1	9
MMAT—Mixed Methods (17)	5	2	1	8
AACODS—Grey Literature (18)	2	1	1	4
**Total**	**28**	**16**	**6**	**50**

NOS, Newcastle–Ottawa Scale; JBI, Joanna Briggs Institute; MMAT, Mixed Methods Appraisal Tool; AACODS, Authority, Accuracy, Coverage, Objectivity, Date, Significance. Risk categories follow standard instrument thresholds. High-risk studies were retained with their methodological constraints weighted during narrative integration. Bold values indicate overall totals.

Of the 50 studies, 56% (*n* = 28) demonstrated low risk of bias, 32% (*n* = 16) raised some concerns, and 12% (*n* = 6) were rated high risk. Individual quality scores are reported in **Supplementary Material S3**.

## 3 Results

### 3.1 Research trends

**Figure 2:**
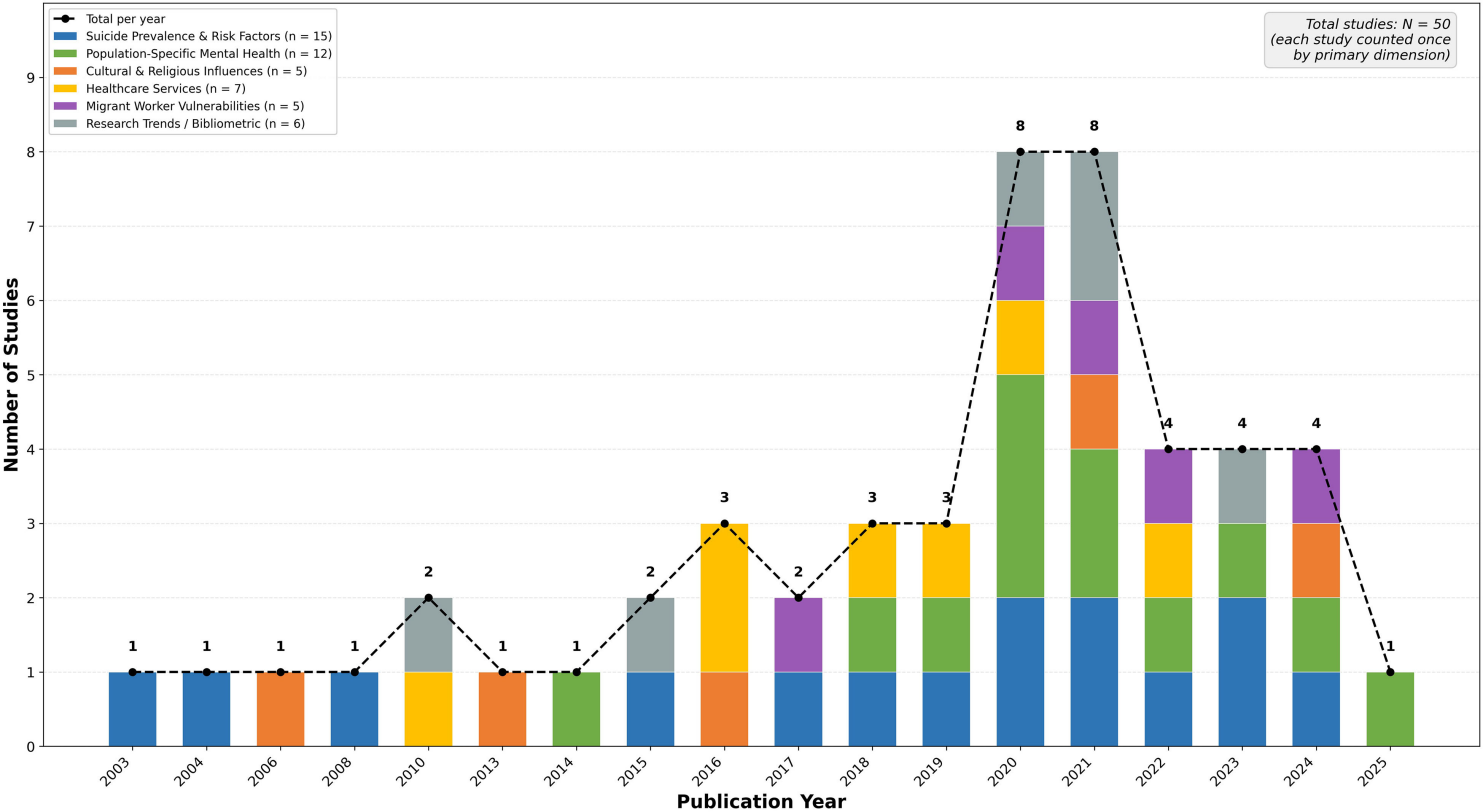
Temporal and thematic distribution of the included studies (N = 50).

Before addressing the four research questions, we first map the temporal and thematic landscape of the evidence base to contextualize the scope and maturity of the available literature. As shown in **Figure 2**, research output expanded substantially after 2015.

Research output expanded substantially after 2015. The 2000–2015 period yielded 16% (*n* = 8) of the total, while 2016–2025 generated 84% (*n* = 42). Publication frequency peaked in 2020 and 2021, with eight studies annually. Across the 50 included studies, each classified by its primary thematic focus, the distribution was as follows: suicide prevalence and risk factors (*n* = 15), population-specific mental health (*n* = 12), healthcare services (*n* = 7), research trends and bibliometric analyses (*n* = 6), cultural and religious influences (*n* = 5), and migrant worker vulnerabilities (*n* = 5).

### 3.2 Suicide prevalence, risk factors, and associated behaviors

Fifteen studies examined the epidemiological and behavioral dimensions of suicidal behavior. **Table 2** provides a taxonomy of these studies.

**Table 2:** Taxonomy of studies on suicide prevalence, risk factors, and associated behaviors (*n* = 15).

Population/context	Focus area	Specific topic	Sources
Adolescents & Youth	Prevalence & Etiology	Parenting styles, school environment, depressive symptoms	Vally & Helmy (19); El-Ghareap Hassan et al. (20)
General Population	Structural Stressors	Social oppression and mental health outcomes	Reber (21)
	Digital Behavior	Internet addiction, insomnia, and suicidal ideation	Kim et al. (22)*; Liu et al. (23)
	Lifestyle Factors	Nicotine dependence and suicidal ideation	Sathian et al. (24)*
Specialized Groups	Occupational Stress	Policing, military stressors, and psychiatric risk	Violanti (25)*; Belik et al. (26)*
Clinical Cohorts	Psychiatric Morbidity	Suicidality in schizophrenia and psychotic disorders	Gooding et al. (27); Amro et al. (28)
	Trauma & Emergency	Self-harm and traumatic injuries	Al-Thani et al. (29)
Regional & Global	Cross-national Trends	Comparative suicide patterns in MENA and globally	Bizri et al. (30)*; Taktak et al. (31)*; Ferguson & Smith (32)*; Qi et al. (33)*

Studies marked with an asterisk (*) are contextual/comparative studies conducted outside the GCC, included for regional benchmarking.

#### 3.2.1 Prevalence and demographic patterns

Reported suicide rates in GCC member states range from 1.5 to 4.2 per 100,000, below global averages (19). These figures likely underestimate true prevalence due to forensic misclassification, legal penalties for attempts, and cultural barriers to disclosure.

Age-specific evidence identifies elevated risk among adolescents (15–19 years) and young adults (20–29 years), particularly in educational settings (19, 20). A consistent gender pattern emerges: males exhibit higher mortality rates, while females report more frequent nonfatal attempts and ideation (21, 29).

#### 3.2.2 Risk factor clusters

Depression, anxiety, and impulsivity are the primary individual-level correlates of suicidal outcomes (20, 27). Among youth, authoritarian and neglectful parenting function as structural risk factors (20). Cigarette smoking shows a dose–response association with suicidal ideation (24). Occupational stress elevates risk in high-exposure professions, as comparative data from law enforcement and military contexts indicate (25, 26). Religiosity is associated with lower rates of death by suicide (3) but religious stigma may simultaneously suppress help-seeking behavior, reducing the likelihood of clinical contact among individuals experiencing suicidal ideation (34).

#### 3.2.3 Behavioral and methodological dimensions

Clinical data from Qatar indicate that nonfatal self-harm predominantly involves medication overdose (approximately 42%) and sharp objects (approximately 31%) (29). Adolescents spending more than five hours daily online face 3.2 times higher odds of suicidal ideation, an association mediated by insomnia (22, 23). The reliance on forensic and administrative records for prevalence estimation remains problematic. Systematic underreporting driven by legal prohibitions and social desirability bias necessitates the integration of psychological autopsy methods and qualitative inquiry.

### 3.3 Mental health of specific populations

Twelve studies examined mental health challenges and suicidal behavior among distinct populations. **Table 3** organizes the relevant studies.

**Table 3:** Taxonomy of studies on the mental health of specific populations (*n* = 12).

Population group	Primary focus	Geographical context	Sources
Migrant Workers	Depression and suicidal ideation	GCC & Malaysia	Sharma et al. (35); Mahat et al. (36)
	Maladaptive coping and returnee suicidality	Ethiopia (GCC returnees)*	Zewdu & Suleyiman (37)*
	Psychological distress and structural risk	Bangladesh*	Kuhn et al. (38)*
Healthcare Professionals	Burnout, depression, and ideation	UAE	Abdulrahman & Farooq (39)
	Depression trajectories	Oman	Al-Houqani & Al-Mukhaini (40)
Vulnerable Groups	Identity loss and social exclusion	Qatar	Al Shamari & O’Hara (41)
	Intimate-partner violence and ideation	Himalayan Region*	Nowshad et al. (42)*
General Population	Systemic discrimination and well-being	Qatar	Abu-Ras et al. (43)
	Major depression and mortality risk	Iraq (Regional)*	Al-Hamzawi et al. (44)*
Regional Burden	Eastern Mediterranean treatment gaps	Eastern Mediterranean*	Charara et al. (45)*
COVID-19 Impact	Migrant-sending community surges	South Asia*	Mia & Griffiths (46)*

*contextual/comparative studies.

#### 3.3.1 Migrant workers

Depression prevalence ranges from 28% to 42% among Nepalese and Ethiopian migrant cohorts (35, 37). Exploitative labor conditions, social isolation, and restricted mobility under the *kafala* system are identified as primary structural correlates. Returnee data from Ethiopia indicate persistent depressive symptoms intertwined with suicidal ideation, exacerbated by substance use (37). Bangladeshi migrants with prior GCC employment report significantly higher psychological burdens than non-migrants (38).

#### 3.3.2 Healthcare professionals

Medical residents in the UAE and Oman have reported burnout rates exceeding 60% and depression prevalence of approximately 38% (39, 40). A systematic review and meta-analysis of healthcare worker mental health across the GCC further reported pooled prevalence estimates of 53% for moderate-to-severe depression and burnout levels reaching 76% (47). These findings suggest that suicidal risk among healthcare professionals in the GCC cannot be understood only through socioeconomic disadvantage. Rather, psychological distress in this group appears to be closely linked to structural workplace pressures, including prolonged working hours, sleep disruption related to extended on-call duties, hierarchical organizational cultures that may limit professional autonomy, and limited institutional pathways for confidential psychological support. Evidence from physicians more broadly suggests that the association between burnout and suicidal ideation operates primarily through depression as a mediating pathway, rather than burnout functioning as an independent predictor (48). Professional stigma may further intensify this risk by discouraging clinicians from seeking help within their own healthcare systems, thereby delaying identification and support for those experiencing severe psychological distress.

#### 3.3.3 Vulnerable groups and regional context

Among individuals facing serious health adversity, patients with spinal cord injuries in Qatar appear to experience depression at substantially higher rates than the general population. Qualitative evidence suggests that suicidal ideation in this group is linked not only to the injury itself, but also to the loss of functional independence, changes in social identity, and perceived reductions in social worth, particularly within social expectations that place high value on self-sufficiency and family role performance (41).

Perceived systemic discrimination represents another pathway to suicidal vulnerability among expatriate populations. Abu-Ras et al. (43) found that experiences of perceived institutional discrimination, especially when combined with limited family support networks, were associated with elevated suicidal ideation among non-citizen residents in Qatar.

The wider regional context also points to persistent gaps in mental healthcare access. Al-Hamzawi et al. (44), drawing on the 2006–2007 Iraq Mental Health Survey, documented substantial treatment gaps for major depressive disorder, although these data predate major changes in conflict exposure and health system capacity across parts of the region. More recent evidence from Iraq indicates that suicidal ideation remains a serious concern among psychiatric populations: Younis et al. (9) found that 29.6% of psychiatric outpatients in Baghdad reported suicidal ideation, with mood disorders, unemployment, and substance use emerging as key correlates. Younis and Lafta (8) also documented continued underreporting and identified young people as a particularly vulnerable group. At the broader Eastern Mediterranean level, Charara et al. (45) estimated treatment gaps exceeding 75%, while the COVID-19 pandemic further exposed these disparities, particularly among South Asian migrant-sending communities where mental health infrastructure was already limited (46).

### 3.4 Cultural and religious influences

Five studies examined the role of cultural norms and religious doctrines in shaping suicidal behavior. **Table 4** presents the taxonomy.

**Table 4:** Taxonomy of studies on cultural and religious influences (*n* = 5).

Dimension	Theme	Geographical context	Sources
Islamic Jurisprudence	Doctrinal distinctions: *haram* vs. *shahada*	Islamic world (Broad)*	Freamon (49)*
Honor Culture	Honor-based violence and gendered risk	MENA region*	El Halabi et al. (50)*
Religious Belief	Spiritual engagement as protective factor	Algeria & Tunisia*	Brik & Aouani (3)*
Stigma & Disclosure	Cultural barriers to psychiatric utilization	Arab populations*	Katz-Sheiban & Eshet (34)*
Epidemiological	Protective effect of active religious practice	Qatar	Ghuloum et al. (51)

* = contextual/comparative studies.

#### 3.4.1 Honor culture and suicidal behavior

Perceived family dishonor—whether realized or anticipated—functions as a proximal determinant of suicidal ideation and attempts, particularly among women (50). In honor-oriented contexts, suicidal acts may be conceptualized as mechanisms for reputation restoration, complicating intervention models premised on individual autonomy.

#### 3.4.2 The religious paradox

Epidemiological data confirm lower suicide mortality among practicing Muslims, indicating that active spiritual engagement serves as a psychosocial buffer (3, 51). However, the doctrinal framing of self-harm as *haram* generates cultural stigma that suppresses disclosure and reduces clinical service utilization (34). The legal distinction between suicide and martyrdom (*shahada*) introduces additional complexity into forensic classification, with contemporary legal systems occasionally navigating blurred boundaries between these categories (49).

This paradox operates differently across the demographic composition of GCC societies. For Muslim citizens and long-term Arab expatriates, the doctrinal prohibition against self-harm may function as an internalized deterrent. At the same time, it may restrict disclosure and help-seeking pathways, as admitting suicidal ideation can carry both spiritual and social stigma. For non-Muslim expatriates, including Hindu, Buddhist, Christian, and other communities represented within the GCC workforce, Islamic normative frameworks may exert influence less through doctrinal internalization than through the legal, institutional, and social environments they help shape. These environments may include punitive or ambiguous legal responses to suicide attempts, limited access to culturally concordant mental health services, and wider social norms that discourage open discussion of psychological distress. Accordingly, the protective dimension of religiosity documented in epidemiological studies (3, 51) should be interpreted primarily in relation to practicing Muslim populations and should not be generalized without qualification to the GCC’s large and religiously diverse expatriate population.

### 3.5 Healthcare and support services

Seven studies evaluated the adequacy and accessibility of suicide prevention services. Notably, four of these (57%) relied on evidence from outside the GCC, reflecting a critical empirical gap in GCC-specific clinical intervention research. **Table 5** presents the taxonomy.

**Table 5:** Taxonomy of studies on healthcare and support services (*n* = 7).

Domain	Focus	Geographical context	Sources
Mental Health Access	Structural and financial barriers	UAE	Hamidi et al. (52); Shahda et al. (53)
Labor Support	Occupational health and migrant prevention	UAE; Nepal*	Atteraya et al. (11)
Military Systems	Peer support and continuity of care	Canada*; UK*	Sareen et al. (54)*; Kapur et al. (55)*
Helpline Services	Evaluation of national helpline	Lebanon*	Zeinoun et al. (56)*
Acute Care Response	Hospital screening and referral protocols	Qatar	Al-Thani et al. (29)

*contextual/comparative studies.

#### 3.5.1 Barriers to service utilization

In Abu Dhabi, patient cost-sharing models for neuropsychiatric care function as financial deterrents for low-income expatriates (52). In Dubai, consultation–liaison services remain underutilized due to limited outpatient capacity and fragmented referral pathways (53). Comparative data from Lebanon identify treatment gaps exceeding 88%, with fewer than 12% of individuals with diagnosable disorders receiving adequate care (56). These findings suggest that despite high per capita health expenditures, GCC states face structural challenges in translating resources into effective coverage for at-risk populations.

#### 3.5.2 Migrant health and the kafala constraint

Occupational health frameworks for migrant workers remain focused on physical safety, systematically excluding mental health screening (11). The *kafala* system, which ties legal status to employer sponsorship, restricts mobility and creates conditions under which suicidal ideation escalates without institutional interception. Multilingual crisis support and confidential reporting independent of employer oversight are absent in most GCC states.

#### 3.5.3 Military systems: analytical proxies

No published data exist on suicide prevention within GCC national armed forces. Canadian and British military programs indicate that peer support networks and sustained post-discharge monitoring reduce ideation (54, 55). The transferability of these models is constrained by regional masculinity norms and rigid organizational hierarchies.

#### 3.5.4 Emergency settings

Hospital data from Qatar identify emergency departments as the primary—and often sole—point of system contact for suicidal individuals. Self-harm presentations account for 3.7% of trauma admissions, with medication overdose as the leading mechanism (29). Fewer than half of these patients receive formal psychiatric evaluation during admission, and only 18% are referred for follow-up care. This pattern represents a significant missed intervention window. This pattern of system contact without behavioral follow-through—where suicidal individuals present to emergency settings but are not connected to sustained behavioral intervention—represents a critical failure in the care pathway.

### 3.6 Migrant worker vulnerabilities

Five studies specifically examined the intersection of labor migration and suicidal behavior. **Table 6** provides the taxonomy.

**Table 6:** Taxonomy of studies on migrant worker vulnerabilities (*n* = 5).

Context	Structural determinant	Health outcome	Geographical context	Sources
Labor Exploitation	*Kafala* system; debt bondage	Suicidal ideation (68% in financial distress)	GCC (multiple)	Fernandez (10)
Financial Distress	Remittance traps; salary withholding	Depression and suicidal ideation	Saudi Arabia	Siddiqui (57)
Sexual Health	Forced exploitation; healthcare exclusion	Depression & suicidal ideation secondary to STD vulnerability	GCC (multiple)	Sohel et al. (58)
Mortality Reporting	Forensic misclassification	Systematic underreporting of suicide deaths	Jordan*	Abder-Rahman et al. (59)*
Return Migration	Post-return adaptive stress	Persistent ideation among returnees	GCC returnees	Sharma et al. (35)

*contextual/comparative studies.

#### 3.6.1 Structural determinants

Financial precariousness, driven by debt bondage and salary withholding, is the dominant structural determinant. Among Nepalese migrants unable to meet financial obligations, 68% reported suicidal ideation (60). Among Bangladeshi women in domestic service, unexplained deaths were frequently suspected as misclassified suicides (57). Forensic data from Jordan indicate that approximately 22% of migrant domestic worker deaths were attributable to suicide (59).

#### 3.6.2 Syndemic interactions

Among undocumented Bangladeshi laborers, STD incidence is 4.3 times higher than among documented counterparts, a disparity that correlates with elevated depression and suicidal ideation secondary to social exclusion (58). The COVID-19 pandemic intensified these risks, as low-income migrants were disproportionately excluded from relief programs and stranded in precarious housing conditions (61).

## 4 Discussion

### 4.1 Underreporting as a defining feature

A recurring finding across the reviewed literature is that official suicide statistics in the GCC are likely to underestimate the true burden. From a forensic psychiatry perspective, this undercount appears to arise through three interconnected mechanisms, each operating at a different point in the surveillance chain. First, punitive or ambiguous legal responses to suicide attempts may discourage self-disclosure by survivors and families. In several GCC jurisdictions, attempted suicide has been addressed through criminal or quasi-criminal frameworks. In Saudi Arabia, Sharia-based legal interpretations have historically treated suicide attempts as offenses against the self, although legal responses may vary across cases (62). In the UAE, earlier penal provisions prescribed imprisonment or fines for suicide attempts, while subsequent diversionary approaches have allowed referral to mental health services in lieu of prosecution in some cases (63). Qatar also retains criminal penalties for attempted suicide (64). These legal and institutional arrangements may create a rational deterrent against disclosure, particularly where families fear investigation, stigma, or legal consequences.

Second, undercounting may occur at the death certification stage through forensic misclassification. Because suicide can carry legal, religious, and social consequences for surviving families, deaths involving ambiguous mechanisms may be more likely to be recorded as accidental or undetermined. This concern is especially relevant in cases involving single-vehicle crashes, drowning, falls from height, or acute poisoning, where intent may be difficult to establish. Evidence from neighboring regional contexts illustrates this risk: Abder-Rahman et al. (59) found that a substantial proportion of migrant domestic worker deaths in Jordan were attributable to suicide, despite some having initially been classified otherwise. Third, cultural stigma may limit disclosure of suicidal ideation and non-fatal suicidal behavior at the population level, leaving much of the non-fatal spectrum outside routine surveillance systems. Taken together, these mechanisms indicate that reported GCC suicide rates of 1.5–4.2 per 100,000 should be interpreted as probable lower-bound estimates rather than definitive prevalence figures (64).

### 4.1 The religious paradox: implications for intervention design

The dual influence of Islamic religiosity represents the most analytically complex finding. Collectivist values and doctrinal prohibitions against self-harm are associated with lower rates of death by suicide (3, 51). Yet the same normative structures generate stigma that obstructs clinical help-seeking (34). This paradox has direct implications for intervention design: programs that leverage religious community structures for mental health outreach may preserve the protective dimension while mitigating the barrier effect. Pilot programs in Kuwait and Saudi Arabia that train religious leaders in mental health first aid represent a promising approach, though formal evaluations remain limited (2). The complication introduced by honor-based normative frameworks—where perceived family dishonor precipitates gendered suicidal behavior—further underscores the need for culturally calibrated intervention models (50).

### 4.3 Structural determinants of vulnerability among at-risk populations

The evidence identifies three population clusters with distinct but overlapping vulnerability profiles. Migrant workers face the most severe structural disadvantage: the *kafala* system, remittance pressures, and institutional exclusion from healthcare create conditions under which suicidal ideation escalates without clinical interception. The 68% ideation rate among financially distressed migrants (60) reflects systemic labor exploitation that demands policy-level reform, not individual clinical intervention. The syndemic interaction between infectious disease burden, institutional neglect, and mental health deterioration (58) reinforces that structural change is a prerequisite for effective prevention in this group.

Healthcare professionals constitute a second at-risk cluster. Burnout rates exceeding 60% and depression prevalence of approximately 38% among medical residents in the UAE and Oman (39, 40), together with meta-analytic evidence indicating burnout prevalence as high as 76% across GCC healthcare settings (47), point to a substantial burden of psychological distress within this professional group. These patterns suggest that suicidal vulnerability among healthcare professionals should not be interpreted solely as an individual clinical problem, but also in relation to the structural organization of medical work. Relevant pressures include prolonged working hours, sleep disruption linked to extended on-call duties, hierarchical workplace cultures that may discourage disclosure of distress, and limited institutional pathways for confidential psychological support. Professional stigma may further compound these pressures by discouraging clinicians from seeking help within their own healthcare systems, thereby delaying identification and support for those experiencing severe psychological distress.

A third cluster comprises individuals facing health adversity and social exclusion. Patients with spinal cord injuries experience depression at rates triple that of the general population, with qualitative evidence linking identity loss to suicidal ideation (41). Expatriates experiencing systemic discrimination report elevated ideation, particularly when familial support is absent (43). Notably, the evidence base on suicidal behavior among GCC nationals remains thin; the structural determinants specific to citizen populations—including youth unemployment, rapid value transitions associated with economic diversification programs, and family-related pressures—are largely unexamined and represent a priority gap for future research.

The paucity of data on GCC nationals warrants explicit acknowledgment. Although this review focuses on suicidal behavior in GCC countries, much of the available evidence concerns non-citizen populations, including migrant workers, expatriate professionals, and clinical cohorts in which nationality is either mixed or not clearly specified. As a result, the structural determinants specific to citizen populations remain insufficiently examined. Potentially relevant factors—such as youth unemployment, pressures associated with rapid socioeconomic transformation, intergenerational value tensions, marriage and family-formation expectations, and changing notions of national belonging—are rarely assessed empirically in the current literature. This is not simply a minor gap in coverage; it limits the extent to which the review can draw conclusions about suicidal behavior among GCC citizens specifically. The evidence is therefore stronger for characterizing suicidal behavior among populations living in GCC countries than for explaining suicidal behavior among nationals of GCC states.

### 4.4 Institutional fragmentation and missed windows

The healthcare services evidence reveals a paradox of expenditure without coverage. Despite high per capita health spending, GCC states demonstrate treatment gaps comparable to lower-income regional neighbors (45). Emergency departments function as the primary contact point for suicidal individuals, yet fewer than half of self-harm patients receive psychiatric evaluation (29). This pattern—clinical contact without systematic risk assessment—represents the most immediately actionable finding for health system reform. The near-total absence of data on military suicide prevention in GCC armed forces constitutes a separate but critical research gap.

### 4.5 Theoretical implications

The empirical patterns identified in this review are consistent with Durkheimian concepts of weakened social integration and normative regulation. Rapid socioeconomic modernization in the GCC may contribute to selective forms of normative fragmentation, whereby traditional sources of social regulation weaken before alternative forms of social consensus become fully established. This dynamic may be particularly relevant for groups navigating tensions between inherited social constraints and emerging modern aspirations, including young adults, migrant workers, and women.

However, a Durkheimian framework alone is insufficient. Structural stressors do not translate into suicidal behavior uniformly across exposed populations; most individuals subjected to labor exploitation, social isolation, or normative uncertainty do not develop suicidal ideation. The stress–diathesis model of suicidal behavior (48) therefore provides a useful clinical complement. It conceptualizes suicidal behavior as arising from the interaction between environmental stressors and pre-existing vulnerability, including psychiatric morbidity such as major depression, psychotic disorders, and substance use disorders (65); neurobehavioral traits such as impulsivity and aggression dysregulation; and cognitive patterns such as hopelessness and cognitive rigidity.

In the GCC context, the structural conditions documented in this review—labor exploitation, institutional exclusion, forensic misclassification, and stigma-related barriers to care—may be conceptualized as stressors, while the psychiatric and psychological conditions identified across the included studies represent elements of individual vulnerability. These include depression among migrant cohorts, high burnout among medical residents, and elevated depression among patients with spinal cord injuries. Integrating these perspectives produces a more balanced explanatory model: suicidal behavior in GCC settings can be understood partly as a Durkheimian social fact shaped by labor systems, legal frameworks, and normative ambiguity, while its expression in particular individuals is mediated by psychiatric and psychological vulnerability.

This combined framework has direct implications for prevention. Structural reforms are needed to reduce exposure to preventable stressors, including stronger labor protections, clearer non-punitive legal responses to suicidal behavior, and better integration of mental health services. At the same time, clinical interventions remain essential for identifying and treating the conditions that heighten individual vulnerability, including depression, substance use disorders, and acute suicidal crises. The reviewed evidence therefore supports an integrated prevention model rather than a choice between structural and clinical approaches.

## 5 Limitations

This review is subject to several constraints. The English-language restriction likely excluded relevant Arabic-language scholarship that may have provided a richer cultural context. The inclusion of 16 contextual/comparative studies, while clearly marked, limits the geographic specificity of certain conclusions. The healthcare services dimension warrants particular caution, as 57% of its evidence relies on international proxies. The cultural and religious influences dimension similarly draws predominantly on non-GCC evidence (four of five studies). The evidence base on suicidal behavior among GCC nationals is notably underdeveloped; structural determinants specific to citizen populations remain largely unexamined. The predominance of cross-sectional designs and small samples precludes causal inference. Data extraction was performed by a primary reviewer with secondary verification rather than independent dual extraction. Geographic coverage is uneven, with the UAE and Saudi Arabia disproportionately represented, while Bahrain, Kuwait, and Oman are underrepresented. The use of Google Scholar as a supplementary database broadens coverage but introduces variability in search reproducibility. The heterogeneity in study designs precluded meta-analysis; narrative synthesis was adopted as the appropriate alternative.

Finally, the evidence base does not adequately distinguish between populations residing in GCC countries and GCC nationals. Much of the available research concerns migrant workers, expatriates, or clinical samples with mixed or unspecified nationality. Consequently, this review can more confidently characterize suicidal behavior among populations living in GCC states than among GCC nationals specifically. Potentially relevant citizen-specific pressures, including youth unemployment, rapid socioeconomic and value transitions, and family-related expectations, remain insufficiently studied and should be treated as a priority for future research.

## 6 Conclusions

This review identifies four core findings corresponding to its research questions. First, reported suicide rates of 1.5–4.2 per 100,000 in the GCC represent floor estimates, systematically suppressed by behavioral avoidance of disclosure, institutional misclassification, and legal deterrence of reporting. Second, Islamic religiosity functions as both a protective factor against death by suicide and a structural barrier to help-seeking, creating a paradox in which the populations most in need of intervention are the least likely to access it. Third, migrant workers constitute the highest-risk group due to the syndemic interaction of kafala-driven labor exploitation, financial distress, social isolation, and healthcare exclusion—structural conditions that elevate suicidal ideation while simultaneously blocking pathways to care. Fourth, healthcare and support services remain fragmented, with emergency departments functioning as the primary point of contact and systematic suicide risk screening absent from occupational, primary care, and community-based settings.

These findings carry direct implications for behavioral intervention design. Culturally calibrated prevention strategies must leverage the protective dimensions of religious community belonging while dismantling stigma-driven barriers to disclosure. Training religious leaders as gatekeepers for suicide risk identification, developing Arabic-language screening instruments that normalize help-seeking, and embedding routine mental health assessment within labor welfare frameworks represent actionable and evidence-informed pathways. The complete absence of behavioral intervention trials in the GCC constitutes the most critical gap in the current evidence base.

Future research should prioritize experimental and quasi-experimental designs testing culturally adapted interventions, longitudinal cohort studies tracking the behavioral impacts of rapid socioeconomic transformation, country-specific investigations across all six GCC states to address the current geographic imbalance, and qualitative research exploring the lived experience of suicidal ideation within collectivist social structures.

In summary, the reviewed evidence points to five priorities for policy and practice. First, GCC health and legal systems should move toward non-punitive responses to suicide attempts, drawing on diversionary models such as the UAE’s 2020 approach, in order to reduce legal deterrents to help-seeking and improve the accuracy of surveillance. Second, routine mental health screening should be integrated into occupational health and labor welfare frameworks for migrant workers, with safeguards for confidentiality and protection from employment-related repercussions. Third, culturally calibrated gatekeeper training should be developed for religious leaders, community figures, and frontline professionals who are likely to encounter individuals in suicidal distress. Fourth, validated screening instruments should be adapted for Arabic-speaking populations and for major migrant-language groups, with wording that reduces stigma and normalizes help-seeking within collectivist social settings. Fifth, GCC-specific psychiatric liaison protocols should be strengthened in emergency departments to support routine risk assessment, safety planning, and follow-up referral for all self-harm presentations.

## Data Availability

All data generated or analyzed during this study are included in this published article and its **Supplementary Materials**. The review protocol is registered on the Open Science Framework (DOI: 10.17605/OSF.IO/RZXY7).
